# APOL3-LDHA axis related immunity activation and cancer ferroptosis induction

**DOI:** 10.7150/ijbs.83342

**Published:** 2023-02-23

**Authors:** Yuzhao Feng, Yunlu Dai

**Affiliations:** 1 Cancer Centre and Institute of Translational Medicine, Faculty of Health Sciences, University of Macau, Macau SAR 999078, China; 2 MoE Frontiers Science Center for Precision Oncology, University of Macau, Macau SAR 999078, China

With the accumulation and advancement of cancer research, researchers have developed more advanced therapies with higher antitumor efficiency and lower side effect, some of which have been approved in clinical application and reach satisfying prognosis in some types of cancer. Nevertheless, cancer progression is a complex process that one type of cancer can be classified into different molecular subtypes based on biomarkers. Even appear morphologically similar, cancer in different subtypes can have dramatically different clinical features and respond variably to therapies 1. Current therapies could cure limited cancer subtypes, mostly in early-to-middle stage. After repeated treatment, some cancer cells may gain resistance ability against drugs or immune reagents. Hence, it's important to find suitable biomarkers for each subtype for distinction and explore the molecular mechanism underlying how to reverse drug resistance and eliminate cancer with the highest efficiency and most suitable therapy.

In this study, Lv et al. identified a significant modulator APOL3 and its negatively regulated downstream protein LDHA, which was named as APOL3-LDHA axis 2. They claimed that overexpression of APOL3 is positively correlated with promoting ferroptosis and CD8+ T cell infiltration at tumor sites and validated that overexpression of APOL3 can be combined with RSL3, a ferroptosis inducer (FIN) and PD-1, an immune checkpoint inhibitor (ICI) to increase cancer sensitivity to ferroptosis and treat immunotherapy resistant colorectal cancer (CRC). This finding is clinically significant because low expression of APLO3 is associated with scarce infiltration of CD8+ T cells and poor prognosis in CRC patients.

The highlight of this paper is to connect APOL3-LDHA axis to ferroptosis and immunotherapy and validated the possibility to utilize APOL3 as a novel biomarker for ferroptosis induction efficiency. Ferroptosis is an iron-dependent form of programmed cell death driven by lipid peroxidation on cell membrane, coined by Dixon et al. in 2012 3. It has sparked great interest in modern cancer research because of its unique mechanism and tumor nature. The metabolic pattern of tumor cells endows them with ferroptosis vulnerability, which might be the potential target in targeting therapy in certain cancer types 4. Furthermore, FINs have been included in some conventional therapies, including chemotherapy and immunotherapy. On the one hand, the induction of ferroptosis in tumor cells have been demonstrated to reverse drug resistance and release DAMPs (E.g. HMGB1), which initiates inflammation and recruits immune cells to TME 5. On the other hand, the conventional therapies could activate cytotoxic T cells and the released cytokines (E.g. IFN-γ) induce tumor ferroptosis. Together, the synergistic effect could potentiate antitumor immunity and restrict tumor progression (Figure 1). Nonetheless, some “smart” cancer cells developed ferroptosis defense systems to survive in high oxidative stress environment and evade attacks from immune system 4. How to disrupt these defense systems and boost cancer sensitivity to ferroptosis remains a challenge. This paper provided a useful strategy to regulate ferroptosis sensitivity by overexpression of APOL3, promoting CD8+ T cell infiltration and IFN-γ release under the assistance of FINs and ICIs. Also, Lv et al. pointed out that APOL3 may serve as a predictive biomarker for ferroptosis-based therapy assessment in CRC treatment, which could be a reference for patients on therapy selection.

Lv et al. focused on verifying the promoted antitumor ability by facilitating ferroptosis, however, the authors mentioned little about its dual function in antitumor immunity, which is a non-negligible concern in the substantial application. Literally, release of DAMPs from ferroptotic cells could activate antitumor immunity. Also, the recruited immune cells such as CD8+ T cells and some T helper cells are susceptible to ferroptosis, which compromises antitumor immunity 6. Additionally, high level of ROS in ferroptotic cells may lead to immunosuppressive TME and dampen the cytotoxic capacity of CD8 T cells 7. How to balance the activation of ferroptosis in tumor cells and immune cells while maintaining significant therapeutic effect remains a critical obstacle. Future prospective on the complicated relationship between ferroptosis and antitumor immunity would be the finishing touch to broaden the horizon of this discovery.

## Figures and Tables

**Figure 1:**
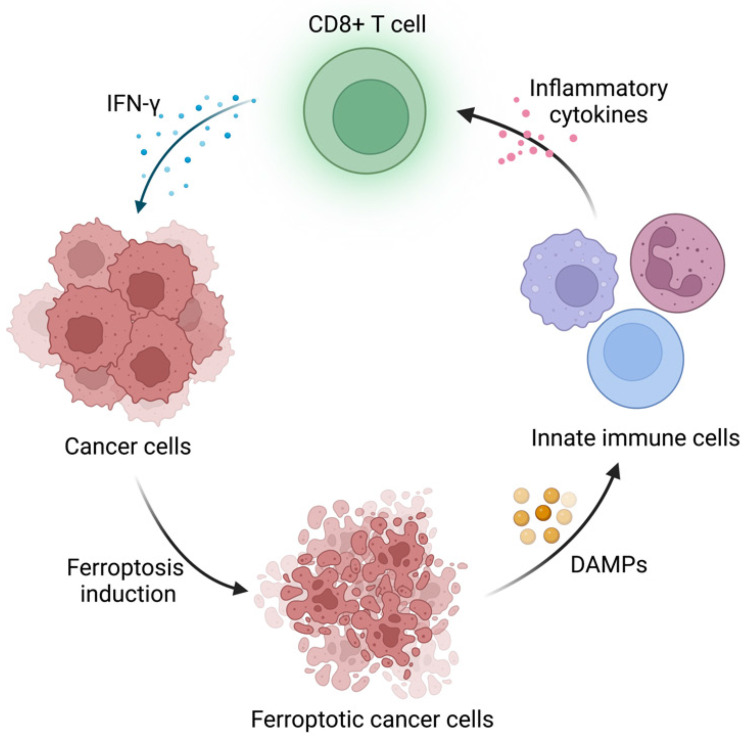
Schematic illustration of the interaction between immune cells and ferroptosis induction in cancer cells. Created with BioRender.com.

## Abbreviations

APOL3: apolipoprotein L3
LDHA: L-lactate dehydrogenase A
ICI: immune checkpoint inhibitor
CRC: colorectal cancer
ROS: reactive oxygen species
FIN: ferroptosis inducer
DAMPs: damage-associated molecular patterns
HMGB1: high mobility group protein B1
TME: tumor microenvironment
IFN-γ: interferon-γ
